# Genetic Associations of *TCF7L2* (*rs7903146*) and *PPARG* (*rs1801282*) with Prediabetes in the Ethnic Kazakh Population

**DOI:** 10.3390/diagnostics14242769

**Published:** 2024-12-10

**Authors:** Azhar Dyussupova, Gulnara Svyatova, Galina Berezina, Altay Dyussupov, Bauyrzhan Omarkulov, Anastassiya Dzharmukhametova, Oxana Yurkovskaya, Venera Akhmetova, Asylzhan Dyussupova

**Affiliations:** 1 Department of General Medical Practice, Semey Medical University, Semey 071400, Kazakhstan; altay.dyusupov@smu.edu.kz (A.D.); anastasiya.dzharmukhametova@smu.edu.kz (A.D.); oksana.yurkovskaya@smu.edu.kz (O.Y.); venera_axmetova@bk.ru (V.A.); assylzhandyussupova@yandex.ru (A.D.); 2 Laboratory of Republican Medical Genetic Consultation, Scientific Center of Obstetrics, Gynecology and Perinatology, Almaty 050020, Kazakhstan; gsvyatova1@mail.ru (G.S.); gberezina54@mail.ru (G.B.); 3 Department of Public Health and Occupational Health, NCJSC Medical University of Karaganda, Karaganda 100012, Kazakhstan; omarculov@qmu.kz

**Keywords:** prediabetes, genetic polymorphisms, *rs7903146*, *rs1801282*, ethnic Kazakhs

## Abstract

Background: This study aims to investigate the genetic contribution of polymorphic variants of the *TCF7L2* (*rs7903146*) and *PPARG* (*rs1801282*) genes to the risk of developing prediabetes in individuals of Kazakh ethnicity. Materials and Methods: This was a case-control study involving 200 cases with prediabetes and 200 prediabetes-free controls, aged 16–60 years (*n* = 400). Real-time polymerase chain reaction on a StepOnePlus instrument (Applied Biosystems, USA), employing the TaqMan method for site-specific amplification and genotyping of the *TCF7L2* (*rs7903146*) and *PPARG* (*rs1801282*) genes was used. Results: Patients with prediabetes had a higher birth weight, increased BMI, larger waist and hip circumferences, and a higher waist-to-hip ratio compared to healthy patients in the control group. There was a significant increase in the risk of developing prediabetes for both the *rs1801282* polymorphism of the *PPARG* gene and the *rs7903146* polymorphism of the *TCF7L2* gene. The risk was 9.8 times higher in carriers of the GG genotype of *PPARG* (*rs1801282*) (OR = 9.769, 95% CI: 2.124–44.922, *p* = 0.003) and 10.7 times higher for carriers of the TT genotype of *TCF7L2* (*rs7903146*) (OR = 10.731, 95% CI: 1.309–87.939, *p* < 0.001). Conclusions: These findings highlight the need for tailored early screening and preventive strategies for prediabetes in the Kazakh population, focusing on individuals with high-risk genotypes. Such efforts could improve targeted interventions and reduce the burden of prediabetes. Future research should adopt a longitudinal design, include diverse ethnic groups, and investigate additional genetic markers to provide a more comprehensive understanding of the genetic underpinnings of prediabetes.

## 1. Introduction

The global obesity epidemic is driving a rapid increase in the prevalence of cardiometabolic disorders, including cardiovascular disease, type 2 diabetes (T2DM), and prediabetes [1,2]. In recent decades, the prevalence of T2DM has escalated significantly, affecting 6.28% of the world’s population and representing a serious public health concern [3]. According to the International Diabetes Federation, the estimated prevalence of T2DM in Kazakhstan among adults is 717,500 cases, or 6.2% of the population [4]. As of 2019, there were 443,776 officially registered T2DM patients in Kazakhstan, marking a 1.7-fold increase compared to 2014. Notably, Kazakh ethnicity has been associated with higher mortality rates in this patient category, among other factors [5].

Prediabetes is a borderline state between normal glucose levels and T2DM, where manifestations of insulin resistance have not yet progressed to the disease stage and can be reversed through timely intervention. Prediabetes is considered a high-risk condition for developing diabetes, with an annual conversion rate of 5–10% [6]. Research in predicting the development of prediabetes is highly relevant not only because of its significant risk of progressing to T2DM but also due to its association with several negative health outcomes, including chronic renal failure, nephropathy, neuropathy, retinopathy, macrovascular diseases, and an increased risk of stroke [6]. The exact causes of prediabetes have not been fully elucidated, but it is believed that carbohydrate and lipid metabolism disorders, obesity, lifestyle, physical activity, nutrition, and environmental factors play significant roles. However, hereditary factors are considered decisive for its occurrence and development [7].

The pathogenesis of T2DM involves factors such as insulin resistance, impaired insulin secretion, increased hepatic glucose production, a sedentary lifestyle, and excessive calorie intake leading to obesity. These lifestyle factors exacerbate genetically determined insulin resistance [8].

Numerous genes have been investigated concerning both T2DM and prediabetes, including *PPARG* (*peroxisome proliferator-activated receptor gamma*, *rs1801282*) and *TCF7L2* (*transcription factor 7-like 2*, *rs7903146*) [9]. *PPARG* (*rs1801282*) has been consistently shown to be associated with insulin resistance and glucose metabolism, with its unfavorable allele linked to impaired fatty acid metabolism and dysfunctional PPARγ2 receptor activity. This pathway is particularly relevant in the context of T2DM development, where disturbances in lipid metabolism are key contributors [10]. The *PPARG* gene plays a crucial role in metabolic regulation and has been extensively studied for its association with type 2 diabetes and obesity. The *rs1801282* single nucleotide variant (SNV) of the *PPARG* gene involves allelic changes C>G and C>T. This variant is located on chromosome 3 at positions 3:12351626 (GRCh38) and 3:12393125 (GRCh37), with canonical SPDI nomenclature designated as NC_000003.12:12351625:C:G and NC_000003.12:12351625:C:T. Additional *HGVS* descriptions include NC_000003.12:g.12351626C>G, NC_000003.12:g.12351626C>T, NC_000003.11:g.12393125C>G, NC_000003.11:g.12393125C>T, NG_011749.1:g.68777C>G, NG_011749.1:g.68777C>T, NM_015869.5:c.34C>G, NM_015869.5:c.34C>T, NM_015869.4:c.34C>G, NM_015869.4:c.34C>T, NM_001354668.2:c.34C>G, NM_001354668.2:c.34C>T, NM_001354668.1:c.34C>G, NM_001354668.1:c.34C>T, NM_001374265.1:c.34C>G, and NM_001374265.1:c.34C>T. Functionally, this variant is classified as a missense variant, coding sequence variant, and intron variant. Its clinical significance ranges from benign to likely benign. This variant results in amino acid changes, including p.Pro12Ala and p.Pro12Ser (e.g., NP_056953.2:p.Pro12Ala, NP_056953.2:p.Pro12Ser, NP_001341597.1:p.Pro12Ala, NP_001341597.1:p.Pro12Ser, NP_001361194.1:p.Pro12Ala, and NP_001361194.1:p.Pro12Ser). The functional implications of these changes highlight the importance of this variant in understanding metabolic diseases, particularly in the context of genetic predisposition to T2DM and obesity [11].

The *TCF7L2* gene has been extensively studied for its association with T2DM and related metabolic disorders. The *rs7903146* single nucleotide variant (SNV) of the *TCF7L2* gene involves allelic changes C>G and C>T. This variant is located on chromosome 10 at positions 10:112998590 (GRCh38) and 10:114758349 (GRCh37), with canonical SPDI nomenclature designated as NC_000010.11:112998589:C:G and NC_000010.11:112998589:C:T. Additional HGVS descriptions include NC_000010.11:g.112998590C>G, NC_000010.11:g.112998590C>T, NC_000010.10:g.114758349C>G, NC_000010.10:g.114758349C>T, NG_012631.1:g.53341C>G, NG_012631.1:g.53341C>T, NG_054085.1:g.746C>G, and NG_054085.1:g.746C>T. The *rs7903146* variant is situated in the intron region of the *TCF7L2* gene and is classified as a genic upstream transcript variant and intron variant. Functionally, it is considered a likely risk allele and a risk factor for T2DM and related conditions. The identification and functional characterization of this variant provide insights into the genetic predisposition to metabolic diseases, emphasizing its importance in population-based studies [12]. *TCF7L2* (*rs7903146*) has demonstrated robust associations with insulin secretion and glucose regulation through its impact on pancreatic β-cells, and alterations in this gene are among the strongest genetic risk factors for T2DM in various populations. This variant is especially important due to its role in modulating the insulinotropic effects of GLP-1, which is critical in the early stages of glucose intolerance and prediabetes [13]. Candidate genes were selected based on an analysis of three primary databases. Specifically, the 1000 Genomes Project was consulted, as it provides comprehensive information on human genetic variation and allele frequencies across diverse populations. Also, the Text-mined Hypertension, Obesity, and Diabetes (T-HOD) candidate gene database was consulted to identify genes previously implicated in these conditions [14]. The *CC* genotype of *TCF7L2* (*rs7903146*) has been associated with elevated levels of C-peptide, potentially indicating enhanced β-cell function in the early stages of carbohydrate metabolism disorders [15].

It has been established that the frequencies of polymorphisms associated with the disease vary among ethnic groups, making them specific to a given population. The identified patterns and distribution of genotypes within a population, along with the prediction criteria developed from these findings, enable the assessment of the risk of developing prediabetes and T2DM. This, in turn, facilitates the implementation of timely preventive measures to mitigate these risks [16]. Several international studies have investigated the prevalence of *PPARG* (*rs1801282*) and *TCF7L2* (*rs7903146*) genotypes in individuals with prediabetes belonging to different ethnicities with varying observations [17,18], but there is a lack of studies on the ethnic Kazakh population. Therefore, this study aims to investigate the genetic contribution of polymorphic variants of the *TCF7L2* (*rs7903146*) and *PPARG* (*rs1801282*) genes to the risk of developing prediabetes in individuals of Kazakh ethnicity.

## 2. Materials and Methods

### 2.1. Study Design and Participants

This was a case-control study involving 200 cases with prediabetes and 200 prediabetes-free controls, aged 16–60 years. All subjects were male and female ethnic Kazakhs, enrolled in routine screening for T2DM conducted by Kazakhstan’s Ministry of Health (MoH).

The screening was conducted as part of a grant-funded study supported by the Ministry of Education and Science of the Republic of Kazakhstan for the period 2022–2024. The screening targeted 2500 individuals of Kazakh ethnicity, aged 16 to 60 years, from the Abai and Karaganda regions at primary healthcare facilities. The screening process involved administering questionnaires and collecting data for individual registration cards, including anthropometric, objective, and laboratory data. This process was carried out during 2022–2023. From the 2500 individuals screened, 200 individuals exhibiting signs of prediabetes (case group) and 200 individuals without prediabetes (control group) were randomly selected based on inclusion criteria.

Ethnicity was determined based on self-reported data, with participants identifying as ethnically Kazakh. To ensure accuracy, we also asked participants to provide information about the ethnicity of their parents and grandparents, confirming Kazakh ancestry across at least three generations. Additionally, participants were recruited from regions predominantly inhabited by individuals of Kazakh ethnicity, further supporting the genetic homogeneity of the cohort.

The selection of study cases and controls was based on the World Health Organization (WHO) criteria for intermediate hyperglycemia, defined as fasting plasma glucose (PG) of 6.1–6.9 mmol/L (110–125 mg/dL) and 2 h PG of 7.8–11.0 mmol/L (140–200 mg/dL) following the consumption of 75 g of oral glucose, or a combination of both based on a 2 h oral glucose tolerance test [19].

Demographic data were collected during their visit to the healthcare facility, as well as measurements of weight, height, hip and waist circumference, and systolic and diastolic blood pressure in both arms. Past medical history data were extracted from medical records. Specifically, information was obtained on the following variables: age; sex (the sample proportion by gender corresponded to the random distribution of participants in routine screening); place of birth; weight; height, hip and waist circumference; weight at birth and presence of overweight or underweight during the first seven years of life; presence of arterial hypertension (AH), ischemic heart disease (IHD), myocardial infarction (MI), stroke, and T2DM in first-degree relatives; and as systolic and diastolic blood pressure in both arms.

Exclusion criteria applied were as follows: age under 16 years or over 60 years; smoking and alcohol abuse; established diagnosis of T1DM or T2DM, pregnancy and lactation; presence of secondary obesity, thyroid, adrenal, or ovarian diseases; presence of severe hepatic and renal pathologies, chronic obstructive pulmonary disease, and bronchial asthma; decompensated AH; acute infectious diseases; and malignant neoplasms. The exclusion criteria were carefully selected to minimize confounding factors that could independently influence the risk of prediabetes.

### 2.2. Collection of Blood Samples and DNA Extraction

Peripheral blood samples were collected from the cubital vein of study participants into EDTA-containing vacutainer tubes. Following centrifugation at 1000× *g* for 10 min at room temperature, the leukocyte-rich interphase was transferred to a sterile, nuclease-free microfuge tube. Genomic DNA was extracted using the QIAamp DNA Blood Mini Kit (Qiagen, Hilden, Germany). The purity of the DNA was assessed by measuring the absorbance 260/280 ratio with a NanoDrop 2000c UV-Vis Spectrophotometer (Thermo Scientific, Waltham, MA, USA); DNA was considered pure if the ratio was approximately 1.8. DNA was then suspended in nuclease-free water and stored at a temperature below −40 °C until further analysis.

### 2.3. Genotyping of the rs18012824 and rs7903146 Variants

The genetic studies were conducted using real-time polymerase chain reaction (PCR) on a StepOnePlus instrument (Applied Biosystems, Carlsbad, CA, USA; Life Technologies Holdings Pte Ltd. Singapore 739256 Blk 33 Marsiling Industrial Estate Rd 3 #07-06), employing the TaqMan method for site-specific amplification and genotyping (TaqMan Genotyper Software Version 1.7.1. Design Analysis Software Version 2.8.0, Thermo Fisher Scientific (manufacturer’s address Waltham, MA, USA 02451 168 Third Avenue). The following commercial kits and reagents were used in the study: the TaqMan SNP Genotyping Assay for *rs7903146* (Cat. No. 4351379, 300 reactions, 750 reactions with a 10 µL buffer) and the TaqMan Drug Metabolism Genotyping Reagents for *rs1801282* (Cat. No. 4362691, 150 reactions, 500 reactions with a 5 µL reaction volume), both from Thermo Fisher Scientific (Life Technologies Limited, Paisley Great Britain, 3 Fountain Drive, PA49RF). Additionally, we used the TaqMan Genotyping Master Mix (Cat. No. 4371353, 1 mL), nuclease-free water (Cat. No. AM 9930, 500 µL), and TE buffer, pH 8.0, RNase-free (Cat. No. AM 9849, 500 mL), also from Thermo Fisher Scientific.

Genotyping was performed within a week after DNA extraction. For DNA dilution, TE Buffer pH 8.0 (composition: 10 mM Tris/HCl, 1 mM EDTA) from Invitrogen by Thermo Fisher Scientific, prepared with ultrapure water, was used. The DNA concentration was measured using a NanoDropLite Spectrophotometer (Thermo Scientific Thermo Fisher Scientific, Fitchburg, WI, USA, 5255 Verona Rd). The DNA purity ranged from 1.7 to 2.0, indicating high-quality DNA suitable for genotyping.

During the genotyping process, high-quality DNA samples were clearly clustered on allele discrimination plots. Any outliers that fell outside the clusters were automatically excluded from the analysis. For such cases, DNA extraction was repeated, and the real-time PCR process was performed again. As a quality assurance step, 1% of genotypes were re-verified, ensuring the validity of the results.

### 2.4. Statistical Analysis

Statistical differences between groups for continuous variables were analyzed using the Mann–Whitney U-test; due to the asymmetrical distribution of the data as assessed by the Kolmogorov–Smirnov test and examination of histograms and Q-Q plots. Differences between groups for categorical variables were tested using the Chi-square (χ^2^) test. Logistic regression was employed to analyze the association between demographic, medical, and genetic risk factors and the odds of prediabetes, with the adjusted odds ratio (OR) and 95% confidence intervals (CIs) being calculated. All statistical procedures were performed using SPSS Statistics software, version 26, with a *p*-value of <0.05 considered statistically significant.

### 2.5. Ethics Statement

The study aims and procedures were explained to all participants, and informed consent was obtained prior to enrollment. For participants under 18 years of age, consent was also obtained from one parent. The study was approved by the local ethics committee of the Non-Commercial Joint-Stock Company “Semey Medical University” (Semey, Kazakhstan), minutes #6 (G-041.11.01.03-202r) dated 22 February 2022.

## 3. Results

Table 1 summarizes the characteristics of the study participants, distinguishing between cases and controls. There were no significant differences between sex, place of birth, or median age. However, a significant difference was observed in birth weight, with cases being heavier than healthy controls. Additionally, cases exhibited significantly greater values in weight, height, body mass index (BMI), waist circumference, hip circumference, and waist-to-hip ratio compared to controls, although height was the only parameter where controls had higher values. Furthermore, cases demonstrated higher median systolic blood pressure in both arms and comparable or higher median diastolic blood pressure in both arms. As the groups were categorized based on fasting PG and 2 h PG levels, cases had significantly elevated levels of these parameters relative to controls.

Cases had significantly higher rates of birth weight either exceeding 4000 g or below 2500 g, as well as a higher prevalence of overweight or underweight during the first seven years of life. Additionally, these cases were more likely to have first-degree relatives with overweight, AH, IHD, DM, MI, and stroke (Table 2). These findings highlight the potential influence of early-life factors, such as abnormal birth weight and weight patterns during childhood, as well as a family history of metabolic and cardiovascular conditions, on the risk of developing prediabetes. This underscores the importance of early screening and targeted preventive strategies for individuals with these risk factors.

Table 3 presents the allele and genotype frequencies for the *PPARG* (*rs1801282*) and *TCF7L2* (*rs7903146*) polymorphisms in cases with prediabetes and controls. There were significant differences in the distribution of all alleles and genotypes between the cases and controls.

Medical history variables (birth weight, weight before age seven, and presence of cardiometabolic disorders in first-degree relatives), which were found to differ significantly between cases and controls, were included as covariates in the regression models to adjust for potential confounding effects. Cases with prediabetes were 9.8 times more likely to carry the homozygous GG genotype of *rs1801282* compared to non-prediabetes controls (OR = 9.769, 95% CI: 2.124–44.922, *p* = 0.003). After adjusting for the presence of AH, IHD, DM, MI, and stroke in first-degree relatives, and birth weight greater than 4000 g or less than 2500 g, the likelihood of carrying this genotype increased to 16.7 times (OR = 16.722, 95% CI: 2.844–98.323, *p* = 0.002). Similarly, cases were 10.7 times more likely to carry the homozygous TT genotype of *rs7903146* compared to non-prediabetes controls (OR = 10.731, 95% CI: 1.309–87.939, *p* < 0.001). After adjusting for the same variables, the odds of carrying this genotype increased to 16 times (OR = 16.080, 95% CI: 1.432–180.568, *p* = 0.024) (Table 4).

These findings suggest that individuals carrying these high-risk genotypes may benefit from targeted screening programs. This underscores the importance of early identification to enable preventive interventions, such as lifestyle modifications or closer metabolic monitoring. At the population level, integrating genetic screening for high-risk genotypes into existing prediabetes prevention strategies could help allocate resources more efficiently and reduce the overall burden of diabetes.

## 4. Discussion

This case-control study aimed to investigate the distribution of *TCF7L2* (*rs7903146*) and *PPARG* (*rs1801282*) genotypes in cases with prediabetes and healthy controls of Kazakh ethnicity. By doing so, the study seeks to elucidate the genetic contribution of these polymorphic variants to the risk of developing prediabetes. Among the genes associated with lipid and carbohydrate metabolism disorders, a significant increase in the risk of developing prediabetes was observed for the *rs1801282* polymorphism of the PPARG gene. Similarly, the risk of prediabetes was significantly elevated in carriers of the unfavorable T allele of the *TCF7L2* gene (*rs7903146*). These findings warrant further consideration in the context of previously conducted research.

The inclusion criteria for the study groups were based on Kazakh ethnicity confirmed through three generations, ensuring the participants’ Kazakh ancestry. Populations from Central Asia are underrepresented in the scientific literature regarding the frequency of gene polymorphisms associated with prediabetes. The Republic of Kazakhstan, located in Central Asia, is predominantly inhabited by Kazakhs, who belong to the Turkic ethnic group.

*TCF7L2* is a key component of the Wnt signaling pathway, which is involved in the regulation of various metabolic processes, cellular growth, and differentiation. It plays a crucial role in regulating the expression of several genes essential for glucose and lipid metabolism. Specifically, *TCF7L2* influences genes involved in insulin production, secretion, and sensitivity [20]. In pancreatic beta cells, *TCF7L2* affects insulin production by modulating the expression of genes that respond to glucose levels and other metabolic cues. Through its regulatory roles, *TCF7L2* helps maintain glucose homeostasis by ensuring appropriate insulin secretion and action, which are essential for blood glucose regulation [20]. Variants of *TCF7L2*, such as *rs7903146*, have been linked to impaired insulin secretion [21]. The association of the *rs7903146* genotype with T2DM is well-known and has been identified in many ethnic populations, as evidenced by the meta-analysis of Ding et al. [22].

The carriage of the T allele was found to be associated with almost a twofold increase in impaired glucose tolerance (IGT) in multiethnic obese adolescents, with a higher rate of progression to T2DM [17]. Carriage of the TT genotype at *rs7903146* was associated with a higher progression to T2DM than carriage of the CC genotype in another longitudinal study [23]. Homozygotes for the T allele had over twice the risk of developing IGT in young Finnish adults [24]. These findings are consistent with the results of this study, which showed an even higher association between the carriage of the TT genotype and prediabetes. This stronger association could be attributed to the case-control nature of this study, as opposed to a longitudinal design, and emphasizes the need for a longitudinal study on the Kazakh population.

*PPARG* encodes a nuclear receptor protein that functions as a transcription factor, regulating the expression of various genes involved in glucose and lipid metabolism. Highly expressed in adipose tissue, *PPARG* plays an important role in adipocyte differentiation and function [25]. It enhances insulin sensitivity by promoting the storage of fatty acids in adipose tissue, thereby preventing ectopic fat deposition in other tissues like the liver and muscle, which can lead to insulin resistance. By improving insulin sensitivity, PPARG helps maintain glucose homeostasis, an important factor in preventing the progression from prediabetes to T2DM [26]. Variants of the *PPARG* gene, such as the *rs1801282* polymorphism, have been associated with altered receptor function [27]. The presence of the homozygous G allele (or the GG genotype) is considered unfavorable, as evidenced by a meta-analysis by Li et al., demonstrating a higher risk of obesity, a risk factor for T2DM [28].

The data on the association of *PPARG* (*rs1801282*) with cardiometabolic disorders, including T2DM, across different ethnicities are contradictory. Several studies have established an increased risk of IGT and T2DM in carriers of the GG genotype in Russian [9], Spanish [29], and Indian populations [30]. These findings align with the results of this study, which showed a significantly increased risk of prediabetes in homozygous G allele carriers. However, a meta-analysis by Sarhangi et al., published in 2020, reported a decreased risk of T2DM for carriers of the GG genotype. This protective effect was found to be significant in certain ethnic groups, such as those of European descent (18% reduction), East Asian descent (20% reduction), and Southeast Asian descent (18% reduction) [31]. Given these conflicting results, this study provides new evidence on the genetic associations of *PPARG* (*rs1801282*) with prediabetes in the Kazakh population. It emphasizes the need to account for ethnic variability in genetic research and highlights the importance of conducting further longitudinal studies to elucidate the role of *PPARG* polymorphisms across different populations.

The issue of T2DM in Kazakhstan is likely to rise as the country gradually shifts toward a Westernized lifestyle, being a part of the global community. This transition is associated with more sedentary behavior and the spread of carbohydrate-rich diets, making not just T2DM but also other non-communicable diseases more common [32]. In this regard, Kazakhstan is not different from other countries in the region, some of which have already witnessed a rise in T2DM cases [33]. This poses significant public health challenges and underlines the need for developing targeted preventive strategies. Understanding the genetic factors associated with prediabetes could provide valuable insights for such strategies, enabling personalized interventions to mitigate the risk of progression to T2DM.

This study has both strengths and limitations. A notable strength is its adequate power and the matching of cases with prediabetes and prediabetes-free controls according to ethnicity, thereby minimizing the influence of ethnic background on genetic associations. Additionally, the study accounts for several potential covariates of prediabetes when analyzing genetic polymorphisms. However, certain limitations must be acknowledged. First, the case-control design inherently affects the generalizability of the findings. While the design is effective for identifying associations between genetic polymorphisms and prediabetes, it does not establish causation and may not fully represent the distribution of genetic variants in the general population. Second, potential selection bias arising from the demographic profile of the sample should be considered. The study focuses exclusively on ethnic Kazakhs from two regions, which might not reflect the genetic and environmental diversity of the entire Kazakh population or other ethnic groups in Kazakhstan. As such, these results may not be generalizable to other populations or even to all subgroups within Kazakhstan. Finally, the focus on only two polymorphisms, *TCF7L2* (*rs7903146*) and *PPARG* (*rs1801282*), while insightful, represents a limitation in comprehensiveness. Prediabetes is a complex, multifactorial condition influenced by multiple genes and environmental factors. Future studies should investigate a broader array of genetic polymorphisms and include more diverse genomic approaches, such as genome-wide association studies (GWAS), to capture the polygenic nature of prediabetes.

Despite these limitations, this study emphasizes the significant role of *TCF7L2* (*rs7903146*) and *PPARG* (*rs1801282*) in prediabetes, suggesting their potential as biomarkers, particularly among individuals of Kazakh ethnicity. These findings provide a basis for developing targeted prevention efforts in Kazakhstan while underscoring the need for further research in other populations and on a wider array of genetic determinants.

## 5. Conclusions

This research emphasizes the necessity of considering genetic diversity when developing targeted prevention and treatment strategies for prediabetes. The findings highlight the importance of early genetic screening, which could identify individuals at higher risk of prediabetes and facilitate timely lifestyle interventions. Future research should be longitudinal in nature and aim to include diverse ethnic groups and a broader range of genetic markers to build a comprehensive understanding of the genetic underpinnings of prediabetes. Such efforts will be crucial in designing effective public health policies and personalized treatment plans that cater to the genetic profiles of different populations.

## Figures and Tables

**Table 1:** Demographic, anthropometric, metabolic, and paraclinical characteristics of the study participants (*n* = 400).

Variables		Study Groups				Test of Statistical Differences	
		Cases (*n* = 200)		Controls (*n* = 200)			
		Median	25th–75th Percentiles	Median	25th–75th Percentiles	U-Test **	*p*-Value
Sex *	Female	150	75.0%	158	79.0%	0.903	0.342
	Male	50	25.0%	42	21.0%		
Place of birth *	Urban	131	65.5%	126	63.3%	0.175	0.676
	Rural	69	34.5%	73	36.7%		
Age, years		31	27–34	29	24–35	−1.664	0.096
Birth weight, g		3400	3200–3600	3200	3100–3500	−3.915	<0.001
Weight, kg		78	69–87	62	54–69	−11.798	<0.001
Height, cm		164	159–170	167	162–171	−2.631	0.009
Body Mass Index		28	26–31	22	20–24	−14.945	<0.001
Waist/Hip ratio		0.84	0.79–0.91	0.78	0.73–0.82	−8.383	<0.001
Waist/Height ratio		0.53	0.48–0.58	0.43	0.41–0.47	−12.496	<0.001
Waist, cm		87	80–95	73	67–80	−11.805	<0.001
Hip, cm		102	96–110	93	89–98	−9.001	<0.001
Systolic blood pressure, right arm		122	110–130	110	110–120	−6.753	<0.001
Diastolic blood pressure, right arm		80	74–87	80	70–80	−4.819	<0.001
Systolic blood pressure, left arm		120	110–130	110	102–120	−7.996	<0.001
Fasting plasma glucose, mmol/L		6.4	6.2–6.5	5.10	4.90–5.30	−17.335	<0.001
2 h plasma glucose, mmol/L		8.50	8.08–8.93	5.70	5.50–6.00	−17.317	<0.001
2 h plasma glucose		5.30	5.00–5.50	5.00	4.70–5.10	−8.325	<0.001

* Test of statistical difference was χ^2^; categorical variables are presented as number and percentage (%); ** Mann–Whitney U-test.

**Table 2:** Birth weight, weight before age seven, and presence of cardiometabolic disorders in first-degree relatives (*n* = 400).

Variables	Study Groups				Test of Statistical Differences	
	Cases (*n* = 200)		Controls (*n* = 200)			
	*n*	%	*n*	%	χ^2^	*p*-Value
Weight at birth >4000 g or <2500 g	26	13.00	9	4.50	9.049	0.003
Presence of overweight or underweight during the first 7 years of life	18	9.00	2	1.00	13.474	<0.001
Presence of overweight in first-degree relatives	39	19.50	131	65.50	86.588	<0.001
Presence of AH, IHD, DM, MI, stroke in first-degree relatives	83	41.50	175	87.50	92.412	<0.001

AH, IHD, DM, MI—arterial hypertension, ischemic heart disease, diabetes mellitus, myocardial infarction.

**Table 3:** Distribution of *PPARG* (*rs1801282*) and *TCF7L2* (*rs7903146*) genotypes and alleles between the study groups (*n* = 400).

Genotypes, Alleles	Study Groups				Test of Statistical Differences	
	Cases (*n* = 200)		Controls (*n* = 200)		χ^2^	*p*-Value
	*n*	%	*n*	%		
*PPARG* (*rs1801282*)						
CC	118	59.00	163	81.50	24.22	<0.001
CG	70	35.00	35	17.50	15.82	<0.001
GG	12	6.00	2	1.00	7.40	0.007
CG + GG	82	41.00	37	18.5	24.22	<0.001
C	306	76.50	361	90.25	27.28	<0.001
G	94	23.50	39	9.75		
*TCF7L2* (*rs7903146*)						
CC	97	48.50	138	69.00	17.34	<0.001
CT	94	47.00	61	30.50	11.47	<0.001
TT	9	4.50	1	0.50	6.56	0.011
CT + TT	103	51.50	62	31.0	17.34	<0.001
C	288	72.00	337	84.25	17.56	<0.001
T	112	28.00	63	15.75		

**Table 4:** Logistic regression analysis of *PPARG* (*rs1801282*) and *TCF7L2* (*rs7903146*) genotypes with respect to the influence of other risk factors (*n* = 400).

Genotypes	Crude OR	95% CI for OR *		*p*-Value	Adjusted OR **	95% CI for OR		*p*-Value
		Lower	Upper			Lower	Upper	
*PPARG rs1801282* (CC)	Reference group				Reference group			
*PPARG rs1801282* (GG)	9.769	2.124	44.922	0.003	16.722	2.844	98.323	0.002
*PPARG rs1801282* (CG)	2.335	1.438	3.791	<0.001	2.167	1.205	3.896	0.010
*TCF7L2 rs7903146* (CC)	Reference group				Reference group			
*TCF7L2 rs7903146* (TT)	10.731	1.309	87.939	0.027	16.080	1.432	180.568	0.024
*TCF7L2 rs7903146* (CT)	2.083	1.356	3.200	<0.001	2.511	1.478	4.266	<0.001

* OR—Odds Ratio; CI—Confidence Interval; ** Adjusted for the presence of AH, IHD, DM, MI, and stroke in first-degree relatives, and birth weight >4000 g or <2500 g.

## Data Availability

The data are available upon request.
